# Supplementary material for: HIVprotI: an integrated web based platform for prediction and design of HIV proteins inhibitors
Source: J Cheminform. 2018 Mar 9;10:12. doi: 10.1186/s13321-018-0266-y (PMC5845081; doi:10.1186/s13321-018-0266-y)
Supplement: Supplementary file 2 — Additional file 2. Source code of HIVProtI web server. [file 13321_2018_266_MOESM2_ESM.zip › HIVprotI_Source-code/hivprotI/weka-3-6-6/doc/weka/classifiers/bayes/net/MarginCalculator.JunctionTreeSeparator.html]

MarginCalculator.JunctionTreeSeparator


---


|  |  |  |
| --- | --- | --- |
| **Overview** \| **Package** \| **Class** \| **Tree** \| **Deprecated** \| **Index** \| **Help** \| **Weka's home** | |  |
| **PREV CLASS**   **NEXT CLASS** | **FRAMES**    **NO FRAMES**     **All Classes** |  |
| SUMMARY: NESTED \| FIELD \| CONSTR \| METHOD | DETAIL: FIELD \| CONSTR \| METHOD |  |


---


## weka.classifiers.bayes.net Class MarginCalculator.JunctionTreeSeparator

```
java.lang.Object
  weka.classifiers.bayes.net.MarginCalculator.JunctionTreeSeparator
```

**All Implemented Interfaces:**: java.io.Serializable, RevisionHandler

**Enclosing class:**: MarginCalculator

---

``` public class MarginCalculator.JunctionTreeSeparator extends java.lang.Object implements java.io.Serializable, RevisionHandler ```

**See Also:**: Serialized Form

---

| **Method Summary** | |
| --- | --- |
| `java.lang.String` | `getRevision()`             Returns the revision string. |
| `double[]` | `update(MarginCalculator.JunctionTreeNode node)`             marginalize junciontTreeNode node over all nodes outside the separator set |
| `void` | `updateFromChild()`             marginalize junciontTreeNode node over all nodes outside the separator set of the child clique |
| `void` | `updateFromParent()`             marginalize junciontTreeNode node over all nodes outside the separator set of the parent clique |

**Methods inherited from class java.lang.Object**

`equals, getClass, hashCode, notify, notifyAll, toString, wait, wait, wait`

**Method Detail**

### updateFromParent

```
public void updateFromParent()
```

:   marginalize junciontTreeNode node over all nodes outside the separator set
    of the parent clique

---


### updateFromChild

```
public void updateFromChild()
```

:   marginalize junciontTreeNode node over all nodes outside the separator set
    of the child clique

---


### update

```
public double[] update(MarginCalculator.JunctionTreeNode node)
```

:   marginalize junciontTreeNode node over all nodes outside the separator set

    :   **Parameters:**: `node:` - one of the neighboring junciont tree nodes of this separator

---


### getRevision

```
public java.lang.String getRevision()
```

:   Returns the revision string.

    :   **Specified by:**: `getRevision` in interface `RevisionHandler`
    :   **Returns:**: the revision


---


|  |  |  |
| --- | --- | --- |
| **Overview** \| **Package** \| **Class** \| **Tree** \| **Deprecated** \| **Index** \| **Help** \| **Weka's home** | |  |
| **PREV CLASS**   **NEXT CLASS** | **FRAMES**    **NO FRAMES**     **All Classes** |  |
| SUMMARY: NESTED \| FIELD \| CONSTR \| METHOD | DETAIL: FIELD \| CONSTR \| METHOD |  |


---
